# Binding of the nuclear ribonucleoprotein family member FUS to RNA prevents R-loop RNA:DNA hybrid structures

**DOI:** 10.1016/j.jbc.2023.105237

**Published:** 2023-09-09

**Authors:** Valery F. Thompson, Daniel R. Wieland, Vivian Mendoza-Leon, Helen I. Janis, Michelle A. Lay, Lucas M. Harrell, Jacob C. Schwartz

**Affiliations:** 1 Department of Pharmacology, University of Arizona, Tucson, Arizona, USA; 2 University of Arizona Cancer Center, Tucson, Arizona, USA; 3 Department of Chemistry and Biochemistry, University of Arizona, Tucson, Arizona, USA

**Keywords:** RNA-binding, R-loop, transcription, FUS, FET protein, RNA:DNA hybrid, RNA, fused in sarcoma, TLS, RNA polymerase

## Abstract

The protein FUS (FUSed in sarcoma) is a metazoan RNA-binding protein that influences RNA production by all three nuclear polymerases. FUS also binds nascent transcripts, RNA processing factors, RNA polymerases, and transcription machinery. Here, we explored the role of FUS binding interactions for activity during transcription. *In vitro* run-off transcription assays revealed FUS-enhanced RNA produced by a non-eukaryote polymerase. The activity also reduced the formation of R-loops between RNA products and their DNA template. Analysis by domain mutation and deletion indicated RNA-binding was required for activity. We interpret that FUS binds and sequesters nascent transcripts to prevent R-loops from forming with nearby DNA. DRIP-seq analysis showed that a knockdown of FUS increased R-loop enrichment near expressed genes. Prevention of R-loops by FUS binding to nascent transcripts has the potential to affect transcription by any RNA polymerase, highlighting the broad impact FUS can have on RNA metabolism in cells and disease.

A prominent member of the heterogeneous nuclear ribonucleoprotein (hnRNP) family is FUS (FUsed in Sarcoma). FUS is best known for gene mutations leading to amyotrophic lateral sclerosis (ALS) or pediatric sarcomas (1, 2). FUS is conserved throughout metazoan species and is among the highest expressed proteins in human tissues (2). FUS has a structure that is largely intrinsically disordered (3). The disordered domains contained in FUS are its low complexity (LC) domain and three arginine and glycine-rich (RGG/RG) domains. Evidence of FUS activity on transcription is reported for all three RNA polymerases in the nucleus (4, 5, 6, 7). FUS binds directly to nascent RNA transcripts, RNA Pol II, RNA Pol III, and other transcription and RNA-processing factors (3, 8, 9). Outside of these, FUS also interacts with mRNA, DNA repair machinery, DNA loops formed during recombination, and snoRNAs (10, 11).

FUS affinity for RNA involves an RNA-recognition motif, Zinc finger domain, and the three disordered RGG/RG domains (12, 13, 14). FUS binds a large number and variety of messenger and noncoding RNAs with a degenerate specificity but prefers G-rich sequences, RNA stem-loops, and more complex RNA structures (13, 14, 15). RNA binding drives FUS to oligomerize into large assemblies, which enhance binding to RNA Pol II and other protein partners (16, 17). In cells, the assembly behavior of FUS is found in protein particles that contain FUS and RNA Pol II and form in a transcription-dependent manner (4, 18). FUS is also enriched near transcription start sites (TSS) of most expressed genes encoding mRNA and prevents phosphorylation of Ser-2 (Ser2P) in the heptad repeat of the C-terminal domain (CTD) in RNA Pol II (6, 19).

We set out to better define the role that binding the RNA polymerase has in FUS activity on transcription. We anticipated direct binding to the polymerase and RNA processing factors would be required for FUS activity (5, 6, 12). However, FUS showed activity on the transcription of a non-eukaryotic RNA polymerase intended to be a negative control. We decided to refocus our study on this unexpected activity observed in the absence of previously known protein:protein interactions.

## Results

### FUS increases RNA yields from T7 Pol transcription

In our previous work, we observed FUS at concentrations found in the cell, >2 μM, greatly increased RNA Pol II transcription from a naked DNA template (4). We asked whether the specific protein-protein interactions FUS makes with RNA Pol II were required for activity. We chose the T7 phage RNA polymerase (T7 Pol) as a negative control that lacked binding sites present in mammalian RNA polymerases (20). Recombinantly expressed FUS with an N-terminal maltose binding protein (MBP) tag and T7 Pol were purified to homogeneity for *in vitro* run-off transcription of a linear DNA template (Fig. S1*A*).

**Figure 1:**
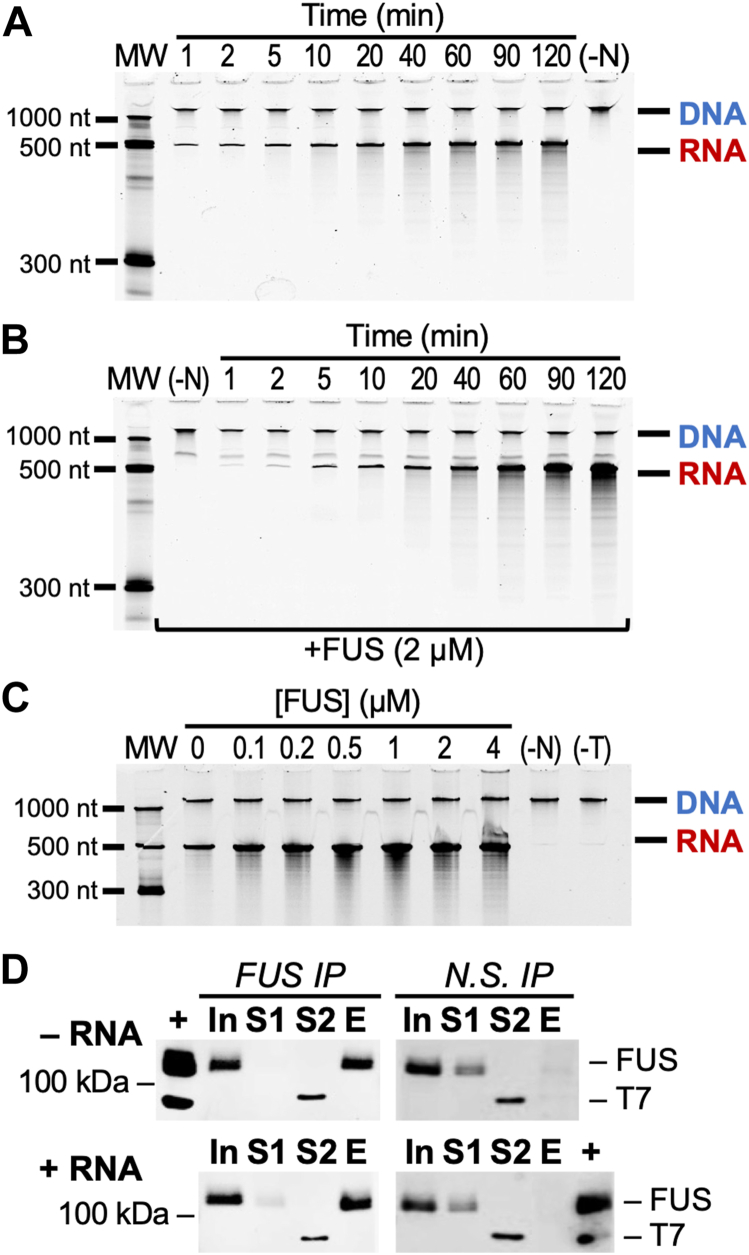
**FUS increases RNA yield of run-off transcription by T7 Pol.***A*, RNA product of T7 Pol run-off transcription seen by urea-PAGE and SYBR staining. The *upper bands* show the DNA template transcribed. *Lower bands* show the increase in RNA product over time. “MW” indicates molecular weight ladder. “(-N)” indicates a negative control without nucleotide triphosphates present. *B*, RNA product of T7 Pol transcription is greater in the presence of FUS (2 μM). *C*, RNA product is increased as a function of FUS concentration. The negative control “(-T)” contains FUS (4 μM), but T7 Pol is not present in the reaction. *D*, Western assays measure FUS and T7 Pol during co-IP with nonspecific IgG or anti-FUS (4H11) antibody. Antibodies for western analysis target FUS (A300-294A) and the 6xHis tag contained in FUS and T7 Pol. Samples shown include input (In), supernatants S1 and S2 after incubation with FUS antibody and T7 Pol respectively, and elution (E) from agarose beads. Assays were performed with RNA present (+RNA) or absent (–RNA). “(+)” lanes contain FUS and T7 Pol for a positive control and molecular weight comparison. See also Fig. S1.

Accumulated products of run-off transcription were observed by PAGE and SYBR staining (Fig. 1*A*). Two stained bands could be seen: a larger band that was confirmed by DNase I digestion to be a linear DNA template (Fig. S1, *B* and *C*), and a smaller band confirmed by RNaseA digestion to be the RNA transcript (Fig. S1*D*). The DNA template detected offered a normalization control for quantitative analysis of PAGE images.

Contrary to our expectation, T7 Pol produced more RNA transcript in the presence of FUS (2 μM) (Figs. 1*B* and S1, *D* and *E*). Titrating buffer constituents present in the assay eliminated these as potential sources of activity (Fig. S1*F*). Increasing concentrations of FUS during T7 Pol transcription increased RNA products observed by PAGE (Figs. 1*C* and S1*G*). Sufficient concentration of FUS produced a shift of RNA to the well, which was reversed by proteinase K treatment to eliminate FUS aggregates formed (Fig. S1*H*). FUS tendency to aggregate has also been noted by previous studies (16, 21).

We investigated whether a direct interaction of FUS and T7 Pol could be observed through co-immunoprecipitation (co-IP). The protocol used for co-IP was successful in detecting FUS binding to protein interactors, including RNA Pol II and III, in previous studies (4, 7, 8, 21). T7 Pol incubated with immobilized FUS remained in the supernatant fraction and undetectable in the elution for negative controls and all replicates but one (N = 4, Figs. 1*D* and S1*I*). Robust binding of T7 Pol could not be provoked by the linear DNA template, or an RNA known to cause FUS to bind RNA Pol II (Figs. 1*D* and S1*I*) (6, 16). We concluded that a protein:protein interaction, like that seen for FUS and RNA Pol II, was unlikely to offer a compelling mechanism for FUS to influence transcription by T7 Pol.

### Requirements for FUS activity on T7 Pol transcription

**Figure 2:**
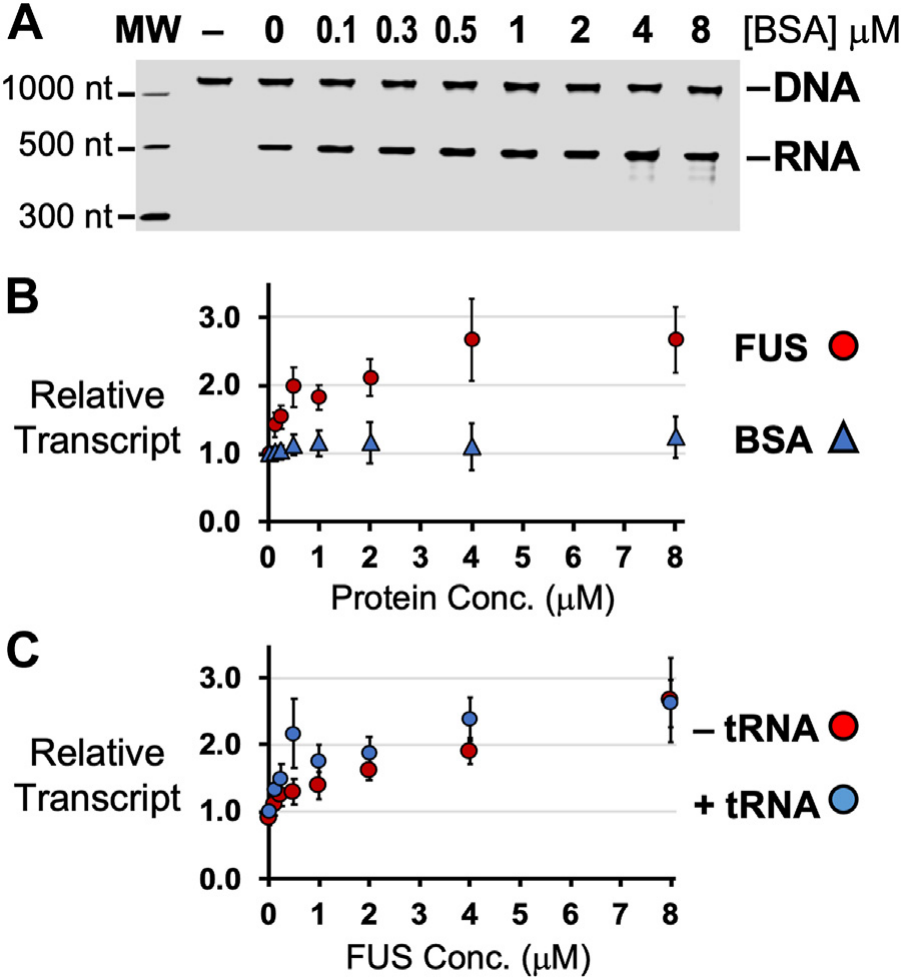
**FUS effects on T7 transcription are specific.***A*, RNA produced by T7 polymerase was not affected by titrating amounts of BSA protein. The negative control samples (−), NTPs were absent in the assay. *B*, RNA quantified by fluorescence also show the presence or lack of increased RNA product caused by FUS or BSA, respectively. *C*, FUS (0.1–8 μM) activity was unaffected with yeast tRNA (6.4 μM) present and quantified by SYBR-stained PAGE analysis to specifically measure RNA produced by run-off transcription. Results are an average of 3 or 4 experiments and relative to transcription without FUS or BSA (see also Fig. S2). Error bars represent ±SEM.

We next considered whether high protein concentrations affected transcription by non-specific molecular crowding. Increases to T7 Pol activity by molecular crowding with glycerol or PEG has been observed by previous studies (22, 23). Activities were compared FUS or a well-folded soluble protein, bovine serum albumin (BSA), present at equal concentration during run-off transcription. BSA effect on RNA production was considerably less than that of FUS (Fig. 2*A*). Fluorescence-based quantification with an RNA-specific dye also detected no activity from BSA (Fig. 2*B*). We also noted FUS activity was diminished after storage at −80 °C, but stored at room temperature, FUS activity remained stable up to 4 weeks, then no activity was seen at 12 weeks (Fig. S2*A*). The lack of effect on transcription by inactive FUS added support to the conclusion that the activity observed was specific.

We considered if RNA in solution might alter FUS activity. Such an effect by RNA or DNA in solution could make the activity of FUS observed during run-off transcription difficult or impossible to observe in a cell, where concentrations of nucleic acids are high. No evidence was found of contaminant bacterial RNA in transcription assays. The 260/280 nm absorbance ratio measured for FUS was <0.6, and no contaminating nucleic acid was seen by SYBR stain (Fig. S1, *B*–*D*, lanes “-N” and “-T”). We also found FUS incubated with RNase was restored to activity by an RNase inhibitor, indicating a contaminating RNA was not required (Fig. S2*B*). We investigated two RNA molecules, a non-specific competitor RNA, yeast tRNA, and an RNA, TET456, previously used to induce FUS binding to RNA Pol II (16). FUS activity was unaffected by tRNA at the same concentration of transcript the assay produced (Fig. 2*C*). Titrating tRNA or TET456 RNA concentrations had no effect on T7 Pol or FUS activity (Fig. S2, *C*–*E*). A repeat of these tests with single-stranded DNA also found the activity of both proteins unaffected (Fig. S2*F*).

### FUS prevents RNA:DNA hybrids formed during transcription

Because FUS is an RNA-binding protein, we considered a mechanism for nascent RNA to affect transcription by binding its complementary template DNA to form an RNA:DNA hybrid. Together with the displaced non-template DNA strand, the structure is termed an R-loop (24). R-loops are found in cells at low but consistent levels, even though they can pose a threat to DNA stability, replication, and transcription (7, 24, 25). For reasons like this, cells possess multiple mechanisms to prevent or remove them (24, 26).

**Figure 3:**
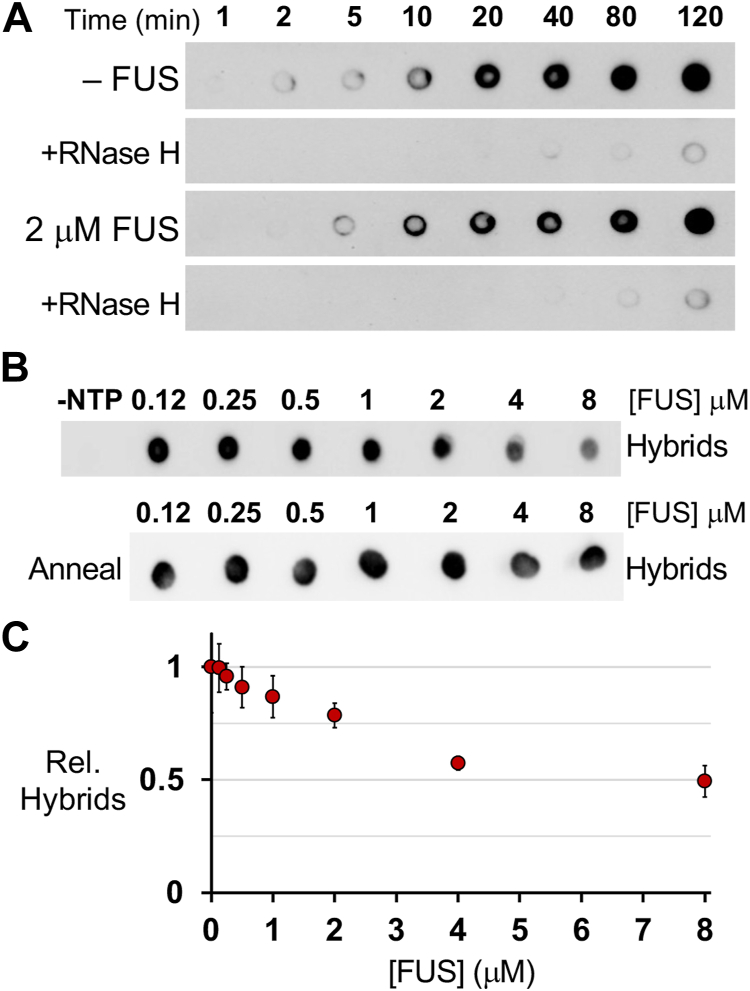
**FUS prevents R-loop formation during transcription.***A*, dot blot assays using the S9.6 antibody detected RNA:DNA hybrids formed during T7 Pol transcription with or without FUS present (2 μM). Incubation of samples with RNaseH (+RNaseH) were used to test antibody specificity. *B*, dot blot assays show RNA:DNA hybrids formed at 20 min with increasing concentrations of FUS protein. The negative control (–NTP) has NTP omitted from the assay. As a positive control, samples were annealed by heating (95 °C) and then cooled to form hybrids. *C*, dot blot assays quantified by densitometry show reduced hybrids formed with FUS present relative to without FUS. Error bars represent ±SEM for 3 experiments.

By dot blot assay using the S9.6 antibody, RNA:DNA hybrids were found to arise early during run-off transcription and their level increased over time (Fig. 3*A*). A nuclease that cleaves RNA:DNA hybrids, RNaseH, could eliminate signals detected, confirming specificity for the S9.6 antibody in this assay (27, 28). Additional controls showed no signal was produced by RNA alone or when NTP or T7 Pol was omitted from the run-off transcription assay (Fig. S3*A*).

The addition of FUS (2 μM) reduced RNA:DNA hybrids observed with the largest difference at 20 to 40 min in the assay (Figs. 3*A* and S3*B*). Increasing concentration of FUS resulted in greater reduction in RNA:DNA hybrids (Fig. 3*B*). Samples were heated then cooled to reveal that RNA transcribed in the presence of FUS retained their full capacity to form an RNA:DNA hybrid (Fig. 3*B*, anneal). Dot blot assays could detect up to a 2-fold reduction in RNA:DNA hybrids resulted from the presence of FUS (Figs. 3*C* and S4*A*).

### Low complexity and RNA-binding domains of FUS contribute to activity

**Figure 4:**
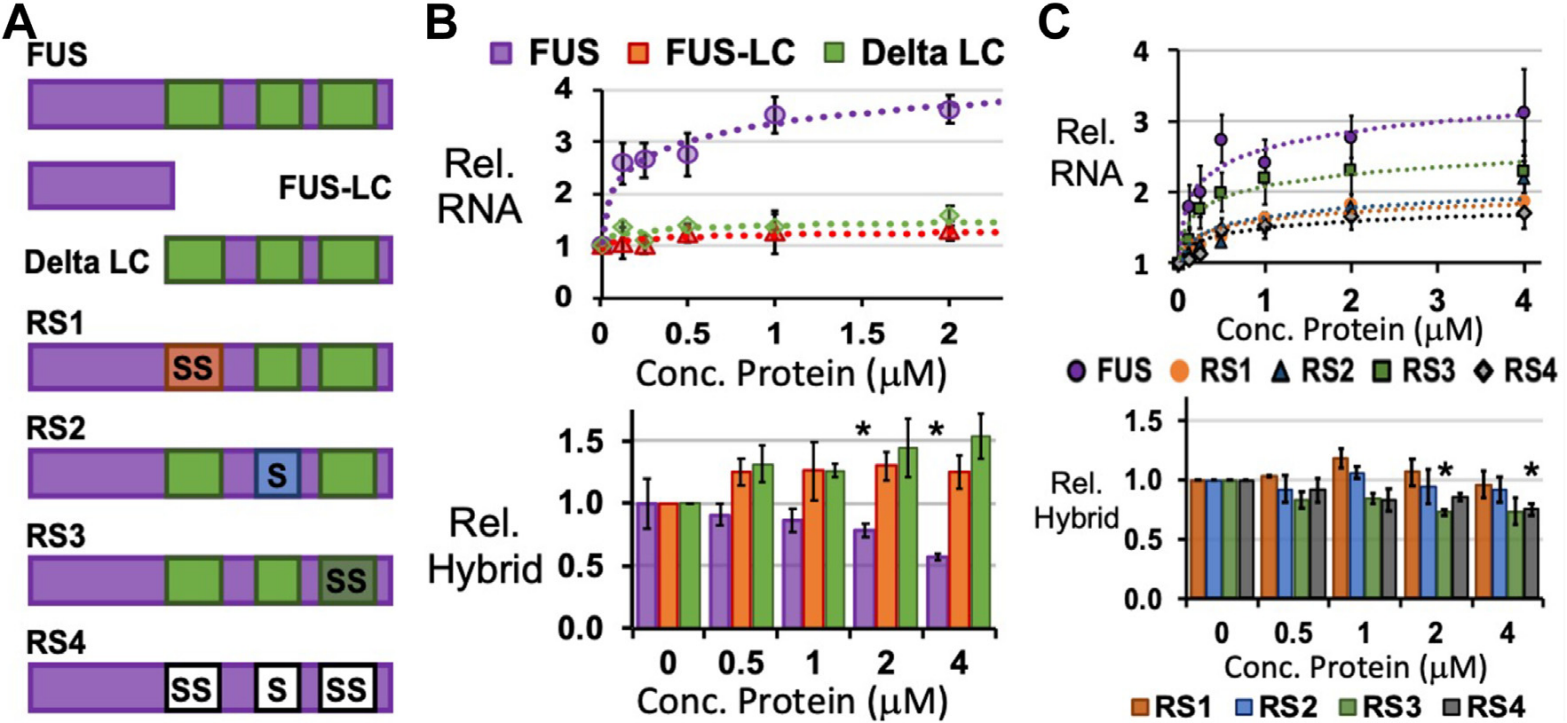
**FUS oligomerization and RNA-binding domains are required to prevent R-loops.***A*, the truncations and amino acid substitutions to FUS are illustrated in relation to its three RGG/RG domains (*green*). Serine residues were substituted for arginine in each RGG/RG domain (RS1, RS2, and RS3) and all RGG/RG domains (RS4). *B*, FUS-LC and Delta-LC truncations were unable to increase RNA synthesis measured by fluorescence-based quantification or reduce RNA:DNA hybrids measured by dot blot assay. *C*, fluorescence-based quantification of RNA products revealed modest increases were achieved by FUS proteins with RS substitutions. The reduction in RNA:DNA hybrids seen by dot blot assays were also modest or insignificant. All results were averaged from 3 or 4 experiments. Error bars represent ±SEM. *Asterisks* (∗) indicate *p* < 0.05, student *t* test assuming equal variances.

We sought to identify FUS domains required for activity on T7 Pol transcription. We investigated truncations of FUS protein that lacked either the LC domain (Delta-LC) or the C-terminal RNA-binding domains (FUS-LC) (Fig. 4*A*). Neither FUS-LC or Delta-LC produced a significant increase in RNA transcript or reduced RNA:DNA hybrids (Figs. 4*B* and S4, *A*–*C*). The lack of activity for Delta-LC and FUS-LC indicated that both self-assembly and RNA-binding, respectively, are required for FUS activity on T7 Pol transcription.

We focused on the role of RNA-binding for activity by substituting serine for arginine residues in one (RS1, RS2, RS3) or all (RS4) of the RGG/RG domains in FUS (Fig. 4*A*). Compared to FUS affinity for RNA, characterization of these substitutions by previous studies found RS1 and RS2 maintained similar affinity, RS3 affinity was reduced by 7-fold, and RS4 affinity was reduced by >30-fold (13). In the run-off transcription assays, RS1 or RS2 increased RNA yields up to 2-fold but did not significantly reduce RNA:DNA hybrids (Figs. 4*C* and S4*D*). RS3 increased T7 Pol transcripts by >twofold, but less than that for FUS. RNA:DNA hybrids were reduced by RS3 a small but significant amount (*p* < 0.05, student *t* test assuming equal variances, Figs. 4*C* and S4*D*). RS4 activity on transcript production matched that of RS1 and RS2, and a small reduction of hybrids could be detected (Figs. 4C and S4*D*).

Finally, inspection by microscopy was performed to assess any self-assembled particles made by FUS or RS proteins. A necessary change from conditions in run-off transcription assays was for proteins to be allowed to incubate at room temperature for 24 h. FUS, RS1, RS2, and RS3 proteins (2 μM) all produced visible particles up to 0.5 μm in diameter, but RS4 yielded no evidence of assemblies (Fig. S4*E*). RS4 particles were not produced by incubation of RS3 and RS4 at 10 μM (Fig. S4*F*). Lastly, FUS and RS4 were tested with protein concentrations up to 100 μM, incubation at room temperature or 4 °C, and addition of RNA, but RS4 did not yield unambiguous particles, while FUS achieved particles ≥1 μm in diameter (Fig. S4, *G* and *H*).

### FUS expression lowers R-loop abundance in human cells

We hypothesized the activity observed for T7 Pol transcription would also be found in human cells. To test this in human HEK293T/17 cells, we used a siRNA approach to knockdown FUS expression and measured R-loop enrichment genome-wide by DRIP next-generation sequencing approach, DRIP-seq, that immunoprecipitates R-loops with the S9.6 antibody (28, 29, 30). Knockdown of FUS protein by an siRNA (siFUS) was determined by Western blot analysis to be >90% relative to control siRNA (SCR) (Fig. S5*A*) (6). Antibody detection of RNA:DNA hybrids and dsDNA in cell lysates was assessed by dot blot assay (Fig. S5*B*). Real-time PCR analysis confirmed that positive control genomic sites, *RPL13A* and *TFPT*, known to contain R-loops was enriched by S9.6 pulldown, relative to a negative control site, *EGR1*. No enrichment was observed for lysates incubated with RNaseH (Fig. S5*C*) (28). Analysis also suggested R-loop enrichment for *RPL13A* and *TFPT* increased in cells with FUS knocked down relative to control (Fig. S5*C*).

**Figure 5:**
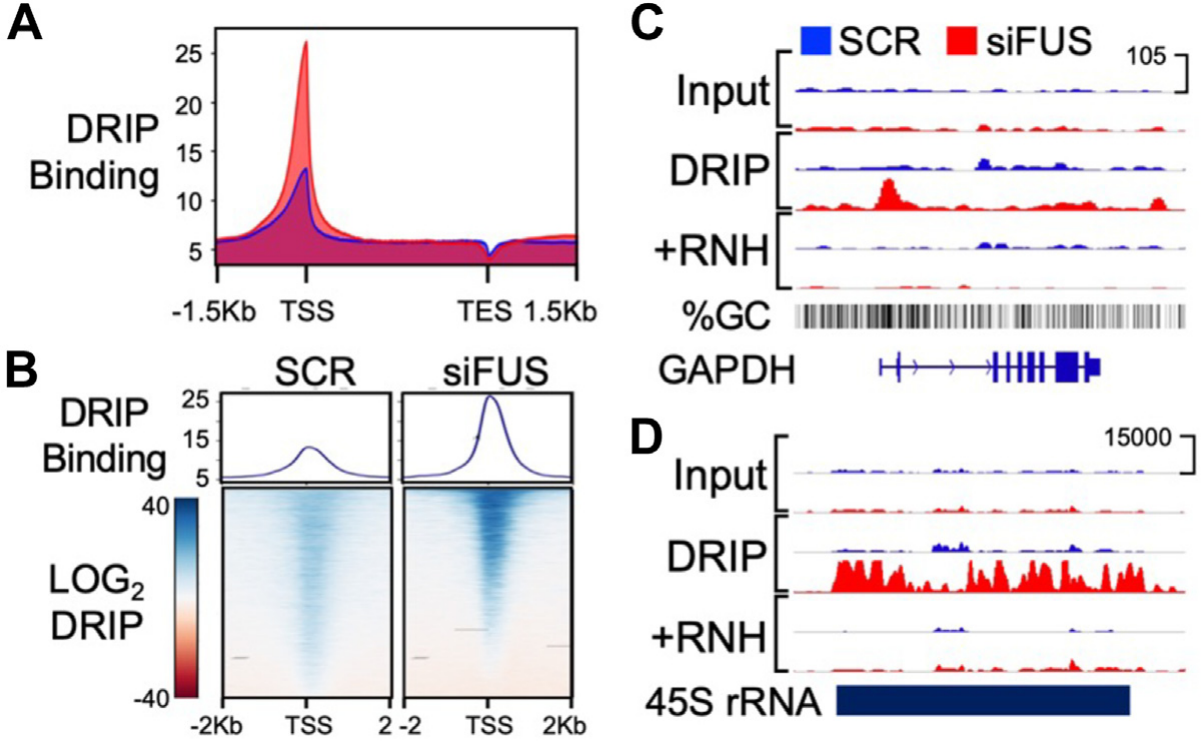
**Knockdown of FUS increases R-loop abundance in human cells.** DRIP-seq was performed on HEK293T/17 cells with knockdown of FUS protein, siFUS, or negative control, SCR. *A*, normalized DRIP-seq signal profiles at human genes were averaged and plotted for siFUS (*red*) or SCR (*blue*) samples. Gene lengths were scaled to align transcription start (TSS) and end (TES) sites. *B*, heat maps show DRIP-seq signals for SCR (*left*) or siFUS (*right*) treatments. *C*, DRIP-seq showing increases to R-loop signal at the *GAPDH* gene promoter resulting from siFUS treatment. *D*, the largest R-loop signals were found at rRNA genes in siFUS-treated samples.

DRIP-seq analysis detected enrichment of R-loops at TSS of expressed genes in SCR-treated cells, and enrichment increased in the siFUS-treated cells (Fig. 5*A*). A 2-fold increase in enrichment was observed at 29% (N = 5260) of protein coding genes (N = 18,188) (Fig. 5*B*). In HEK293T/17 cells, a gene previously found by ChIP-seq and CLIP-seq analysis to be enriched for FUS is *GAPDH* (6, 9). In DRIP-seq analysis, R-loop enrichment near the *GAPDH* promoter increased substantially in siFUS-treated samples relative to the input, SCR-treated, or RNaseH-treated samples (Fig. 5*C*) (6). Though previous ChIP-seq and CLIP-seq studies could only detect FUS enrichment at RNA Pol II genes, DRIP-seq analysis of the FUS knockdown found the largest increase in R-loop enrichment to be at ribosomal RNA (rRNA) genes transcribed by RNA Pol I (Fig. 5*D*) (6, 11, 21, 31, 32).

While an increase in R-loops caused by FUS knockdown was predicted by our T7 run-off transcription studies, the localization of R-loops observed by DRIP-seq were consistent with previous studies of FUS activity in cells. FUS binding specificity to RNA is degenerate but does prefer GC-rich sequences (13, 14, 33). In the FUS knockdown, changes to R-loop enrichment were mostly at GC-rich DNA sequences (Fig. S5*D*). Also, peaks for R-loop enrichment were primarily in intergenic and satellite DNA in SCR-treated cells (N = 5045 peaks), and consistent with published ChIP-seq and CLIP-seq data, peaks in siFUS-treated cells were mostly in protein-coding genes and promoters (N = 7247 peaks) (Fig. S5*E*) (16). DRIP-seq also offered novel evidence of FUS activity, including changes to R-loop enrichment at repetitive DNA features (Fig. S5*F*). Finally, FUS has been shown to have activity on RNA Pol III transcription (5, 34). Inspection of these genes found increased R-loop enrichment at 5S rRNA genes, but not *RMRP*, *RN7SK*, or two tRNA gene clusters on chromosomes 1 and 6 (Fig. S5*G*).

In conclusion, the analysis of DRIP-seq indicated widespread FUS activity preventing R-loops in human HEK293T/17 cells. FUS activity on R-loops was not limited to transcription by RNA Pol I, II, or III. The changes to R-loop enrichment that could be measured by DRIP-seq revealed a close similarity of FUS activity in human cells and that observed during *in vitro* run-off transcription.

## Discussion

Here we explore FUS activity on transcription that involves the potential for RNA transcripts to bind their DNA template, forming an R-loop. Our study followed the unexpected result that FUS increased the RNA yield from T7 Pol transcription. Subsequent analysis determined FUS reduced RNA:DNA hybrids that formed during transcription. Our model is that FUS increases RNA yield by binding nascent transcripts to prevent or delay R-loop formation, which would slow or block the RNA polymerase. In human cells, a reduction of FUS protein increased R-loop abundance. The R-loop enrichment was most prominent at expressed genes, notably those transcribed by RNA Pol I and II. These results indicate FUS bound to nascent RNA transcripts can influence transcription by preventing R-loops.

Our findings indicate that both the self-assembly of the LC domain and the RNA-binding are important to FUS activity on RNA:DNA hybrids. This simple interpretation is made from the loss of activity for the delta-LC and FUS-LC truncations, which retain or lack RNA-binding activity respectively (Fig. 4*B*). Inspection of individual RGG domain contributions did not conclude that one could be mostly responsible for activity. The RS3 protein retained some activity on the amount of transcript produced, but activity on R-loops was indistinguishable from the RS4 protein, which has a >20-fold higher K_D_ for RNA and no activity on product amounts (Fig. 4*C*). On the one hand, binding affinity is measured in steady-state conditions and transcription is a dynamic system. Specifically, the difference in RNA production in the presence of FUS is seen over 2 h of run-off transcription, while the difference in RNA:DNA hybrids is undetectable beyond 40 min. This can indicate effects of non-equilibrium or combined states in transcription and FUS activity (35, 36). A small impact on R-loops early in the assay strongly affects transcription rates over a greater length in time.

Previous studies indicate that FUS can modify transcription by RNA Pol I, II, and III (2, 5, 7, 37). We have added to this list T7 Pol from bacteriophage, which shares no unambiguous sequence similarity to eukaryote RNA polymerases (38). The mechanisms of FUS activity on eukaryote RNA polymerases are complicated (2). FUS binds *in vitro* and *in vivo* the CTD and other subunits of RNA Pol II, and this activity is potentiated by FUS phase separation (1, 3, 16, 17). The LC and RGG3 domains show the greatest affinity for the CTD (3). FUS also binds or modifies the activity of several factors influential to RNA Pol II transcription, including SR proteins, BRD4, CDK9 (P-TEFb), and the U1 snRNP (8, 12, 19, 39, 40). A fusion protein involving the DNA-binding domain of a transcription factor, such as DDIT3 or ERG, and the LC domain of FUS can redirect RNA Pol II activity sufficient to drive Ewing and other sarcomas (1, 41). FUS activity on RNA Pol I and III is less studied but also complex. While binding between FUS and RNA Pol III is reported, FUS interactions with TATA-binding protein (TBP) offer a potent mechanism to alter transcription by RNA Pol I or III (5, 34).

Our run-off transcription results suggest that by preventing R-loops, FUS addresses an obstacle faced by any RNA polymerase during transcription. R-loops have many effects on transcription and chromatin stability besides the simple stalling of the polymerase that we have studied here (24). Previous studies of FUS effects on RNA transcription have emphasized mechanisms by binding to the polymerase directly or indirectly by transcription factor proteins (2, 12, 19, 34). The mechanism described here differs by acting through an RNA interaction and binding to the polymerase is not required (16, 17). Our assays employed T7 Pol, which is unlikely to possess a conserved binding site with human RNA polymerases. Our co-IP assay did not yield evidence of FUS binding to T7 Pol (Fig. 1*D*).

Like FUS activity found in T7 Pol transcription, our DRIP-seq analysis finds endogenous FUS is also involved in preventing R-loops in the cell. At least two studies anticipated FUS would play a role in either prevention or repair of R-loops. First, a study found that DNA damage where FUS was recruited had characteristics of R-loop-induced damage, and damage resulting from a loss of FUS was partly reduced by overexpressed RNASEH1 (7). Second, FUS mutations are known to lead to ALS and studies have correlated the disease to increased levels in R-loops, including a recent study finding FUS was pulled down with R-loops in cell lysates (42, 43, 44). Ewing sarcoma has also been found to be highly enriched for R-loops, which was linked to EWSR1 inactivation by the EWS-FLI1 protein (29). It remains to be tested whether this also occurs when FUS fusion proteins are driving the disease.

The R-loops found at so many RNA Pol II genes after FUS knockdown in HEK293T/17 cells mirrors the enrichment of FUS previously found at promoters for most expressed genes in this cell line (6, 21). As interpreted from the *in vitro* assay results, the simplest explanation for R-loops observed at sites transcribed by RNA Pol I, II, and III is an activity mediated by binding RNA rather than the polymerase (Fig. 5). Ordinarily, increased transcription would favor RNA:DNA hybrid formation, but an RNA-binding protein that prevents R-loops is not unprecedented (45). Failure to recruit snRNPs, SR proteins, and hnRNPs can allow R-loops to form (29, 46, 47). Protection against R-loops by SRSF1 is one example that has been reproduced in run-off transcription by T7 Pol (47). The effect found by DRIP-seq also indicates that a loss of FUS cannot be easily compensated by another RNA-binding protein. This may be due in part to the contribution of the LC domain (Fig. 4*B*).

In conclusion, the ability of FUS to influence transcription can be extended to include preventing R-loops by binding nascent RNA transcripts. FUS and R-loops have complex relationships with transcription and DNA stability. The mechanism we describe would not conflict with those that involve binding the polymerase, transcription factors, or RNA-processing machinery. It is noteworthy that FUS and R-loops are contributors to the same neurodegenerative diseases (7, 43, 48, 49). Since FUS is highly expressed protein in human cells, its activity likely has significant implications to R-loop function in both biology and disease.

## Experimental procedures

### Recombinant protein expression and purification

FUS constructs used in this study are available upon request. All recombinant proteins were expressed and purified from *E. coli* BL21(DE3) cells. All FUS constructs were N-terminally fused to 6× His and MBP to improve purification and solubility. Protein expression was induced after cell growth reached an OD600 of 0.8 and then allowed to continue overnight at 17 °C with shaking (200 rpm). Purification was made using 1 to 2 g of frozen pellets of induced *E. coli*, lysates incubated with Ni-NTA Sepharose beads (Cytiva Lifesciences, 17531802), and eluted in FUS-SEC buffer (1 M urea, 0.25 M KCl, 50 mM Tris-HCl pH 8.0) with 250 mM imidazole and either 1.5 mM β-mercaptoethanol or 1 mM DTT added (4, 13, 15). MBP-FUS concentrations were determined by UV absorption at 260 nm using extinction coefficient of 1.42, and 260/280 nm ratios measured were <0.6.

Plasmids for protein expression of T7(P266L) polymerase fused with a 6xHis tag were provided by A. Berman (University of Pittsburgh) (50). Expression in *E. coli* BL21(DE3) cells and purification were done essentially as previously published (51). Protein expression was induced by 0.5 mM Isopropyl β-D-1-thiogalactopyranoside (IPTG) when transformed cells grew to an OD600 of 0.6 then continued for 3 h at 37 °C. Induced cells stored at −80 °C were thawed, lysed in T7 Buffer (50 mM Tris-HCl pH 8.0, 100 mM NaCl, 5% v/v glycerol, and 5 mM β-mercaptoethanol added just before use) with 1 mM imidazole then incubated with Ni-NTA beads. T7 Buffer with increasing imidazole was used to wash beads 4 times (1 mM imidazole), 2 times (10 mM imidazole), and to elute protein (100 mM imidazole).

Size-exclusion chromatography (SEC) was performed with a 10/300 Superdex 200 column (Cytiva Lifesciences, 17517501) for FUS using FUS-SEC buffer and for T7 Pol with T7-SEC buffer (20 mM Tris-HCl pH 7.5, 100 mM NaCl, 0.1 mM EDTA, and 1 mM DTT). FUS protein was stored at room temperature for up to 4 weeks or until activity appeared lost. T7 Pol activity was assessed by titrating protein into transcription assays as described below. The absence of nuclease activity was confirmed by comparing plasmid DNA and transcribed RNA incubated overnight with or without protein and electrophoresis using a Novex TBE-urea, 6% polyacrylamide gel (Invitrogen, EC68655BOX) and stained with SYBR-Gold (Invitrogen, S11494).

### Nucleic acid preparation and purification

DNA substrates used in T7 transcription assays were prepared from pcDNA3 vector plasmid and the gene inserted for RNA transcription was Flag-FUS (13). Bsu36I restriction enzyme (50 U, NEB, R0524S) was used to linearize plasmid (10 μg) by incubating at 37 °C for a minimum of 2 h in 1× CutSmart buffer (NEB, B7204S). Linearized plasmid was purified by extraction with an equal volume of phenol:chloroform:isoamyl alcohol (25:24:1, pH 6.7/8.0) (Fisher Scientific, BP1752I-100) followed by two additional chloroform extractions (VWR, 97064-680) and ethanol precipitated. Plasmid concentrations were measured by UV absorption (260 nm) using a Biotek Epoch 2 plate reader with a TAKE3 plate. RNA TET456 was provided by T.R. Cech (University of Colorado Boulder) (52). Additional nucleic acids were yeast tRNA (Invitrogen, 15401011) and a commercially synthesized ssDNA (Millipore-Sigma). Nucleic acid sequences can be found in Table S1.

### T7 transcription

Transcription reactions were prepared at room temperature in 20 μl transcription buffer (40 μM Tris-HCl pH 8, 5 mM DTT, 10 mM MgCl_2_) with 2 mM NTP’s (NEB, N0450S) and 40 U/μl RNasin Plus (Promega, N2615). T7 (P266L) polymerase (20) was added to linearized plasmid DNA (25 ng/μl) at 37 °C, incubated for up to 2 h, and halted either by heating to >75 °C or addition of EDTA (12 mM final concentration). To detect RNA products by electrophoresis, samples were heated (90 °C, 5 min) in formamide loading dye (49% formamide, 5 mM EDTA, 0.05% xylene cyanol, 0.05% bromophenol blue) and loaded to a Novex TBE-Urea, 6% polyacrylamide gels (Invitrogen, EC68655BOX) to run in TBE (VWR, 97061-754) at 180 V for 50 min. A ssRNA ladder (NEB, N0364S) was used for molecular weight standards. Gels were stained with SYBR-Gold (Invitrogen, S11494) in TBE and imaged using a Bio-Rad ChemiDoc MP Imaging System. Densitometry was performed with Bio-Rad Image Lab Software v. 6.0.1. The ratio of RNA/DNA was calculated for each sample to control for loading differences. RNA and DNA were also quantified by Quant-iT Assay (Thermo Fisher Scientific, Q333140 and Q33120) according to manufacturer’s instructions.

### Co-immunoprecipitation assay

Recombinant expressed FUS protein (1.3 μg) and anti-FUS (4H11) antibody (11 μg; Santa Cruz Biotechnology, sc-47711) were immobilized by incubating together for 1 h at room temperature in phosphate buffered saline (PBS, pH 7.4) (Sigma, P3813) and 0.1 mg/ml bovine serum albumin protein (VWR, 97062-508), then 1 h with Pierce Protein A/G agarose beads (Thermo Fisher Scientific, 20421). Beads were washed 4 times with 0.1% NP-40 substitute (VWR, 97064-730) in tris-buffered saline (TBS). Beads with FUS were incubated with T7 Pol for 1 h at room temperature with rotation, then washed twice in high salt, twice in TBS with 0.1% NP-40 substitute, and one with TBS. Elution of protein bound to beads was incubated at 95 °C for 10 minutes in 2× NuPAGE LDS sample loading buffer (Life Technologies, NP0008) containing 4% lithium dodecyl-sulfate (LDS) and DTT (100 mM) added. Nucleic acids used were prepared as described earlier and Table S1. Detection of eluted proteins was made by Western blot assay using anti-FUS (A300-294A, Bethyl Laboratories) to identify FUS protein and anti-His (HIS.H8, Novus Biologicals, NBP2-31055) to identify His tags for both FUS and T7 Pol proteins.

### RNA:DNA hybrid dot blot assays

RNA:DNA hybrids were detected and quantified by dot blot assay. From transcription assays, 10% of reactions were spotted on N+ membranes (Fisher Scientific, 45-000-850), dried and UV crosslinked. Blots were blocked with 5% nonfat dried milk (NFDM) in TBS-T, then incubated overnight at 4 °C with an anti-RNA:DNA hybrid antibody S9.6 against RNA:DNA hybrids (1/2000 dilution) in TBS-T with 2.5% NFDM. Blots were washed 5 min 4 times in TBS-T, incubated in secondary antibody (goat anti-mouse IgG-HRP, 1/20,000) for 1 h at RT. Images were taken after incubation with SuperSignal West Pico PLUS (Fisher Pierce, PI34578). Images were acquired with a Bio-Rad ChemiDoc MP Imaging System. Densitometry was performed with Bio-Rad Image Lab Software v. 6.0.1.

### DRIP-seq assay and data analysis

DRIP-seq assays were performed essentially as a previously published protocol (28). HEK293T/17 cells (ATCC, CRL-11268) grown and passaged in DMEM (5% FBS) were transfected by siRNA using RNAiMax (Invitrogen, 13778150) in Optimem (Invitrogen, 31985070). Sequences of the siRNA targeting FUS, and the scramble control are included in Table S1. Between 7 and 9 × 10^6^ cells were harvested 72 h post-siRNA treatment, and cell pellets were frozen in liquid nitrogen. Pellets were later resuspended in 1.6 ml of TE buffer (10 mM Tris-HCl pH 8, 1 mM EDTA) that was added to include 0.6% sodium dodecyl sulfate (SDS) and 60 μg/ml Proteinase K. After Proteinase K digested lysates overnight at 37 °C, DNA was extracted using the MaXtract High Density tubes (Qiagen, 129065). DNA was spooled by glass Pasteur pipette, transferred, then ethanol precipitated.

DNA was digested overnight in TE buffer with 1× NEBuffer 2 (New England Biolabs Inc, #B6002), BSA (95 μg/ml), spermidine (1 mM), and 30 U each of restriction enzymes BsrGl, EcoRI, HindIII, SspI, and XbaI. Restriction digest was confirmed by agarose (0.8% w/v) gel electrophoresis. After restriction enzyme digestions, DNA was extracted by Phase Lock Gel (VWR, 10847-800) per manufacturer instructions. Negative control samples were digested by 40U RNaseH (Fisher Scientific, 50-811-717) per 10 μg DNA. 50 μg of DNA was incubated overnight at 4 °C with 20 μg S9.6 antibody and in 500 μl DRIP buffer (1× TE buffer, 10 mM Na phosphate pH 7, 140 mM NaCl, 0.05% Triton X-100). 5 μg of DNA was kept for input samples. Antibody precipitated with 100 μl Protein A/G beads (Millipore, IP05-1.5ML) was washed twice in 700 μl DRIP buffer. Hybrids were eluted by incubating beads with 300 μl DRIP Elution buffer (50 mM Tris-HCl pH 8, 10 mM EDTA, 0.5% SDS, 0.5 μg/μl Proteinase K) for 45 min at 55 °C, then extracted by Phase Lock Gel. Samples were sonicated for 30 cycles (30 s on/30 s off) follow by ethanol precipitation.

Sample libraries were prepared and sequenced by Novogene Corporation Inc using a NovoSeq6000 with 150 base paired-end reads (8 G raw data per sample). Reads were trimmed using TrimGalore (version 0.6.6) and aligned to GRCh38 using STAR (version_2.7.8a), essentially as previously described. Bam files were processed using the deepTools2.0 suite (version 3.5.1) to normalize to 1× depth (reads per genome coverage) with bamCoverage, merge replicates by bigwigCompare, and tags binned by computeMatrix, which was then used to compute scaling factors and generate heat maps and profiles. Merged bigwig files were converted to bedgraph and then peaks by Macs2 (version 2.1.1.20160309). HOMER (version v4.11.1) was used to calculate GC-content and annotate peaks. Data was visualized, and figures were generated using Integrated Genomics Viewer (version 2.5.0). DRIP-seq data is available (GSE206740) from the Gene Expression Omnibus (https://www.ncbi.nlm.nih.gov/geo/).

## Data availability

DRIP-seq data is available (GSE206740) from the Gene Expression Omnibus (https://www.ncbi.nlm.nih.gov/geo/).

## Supporting information

This article contains supporting information.

## Conflict of interest

The authors declare that they have no conflicts of interest with the contents of this article.
