# Supplementary material for: Binding of the nuclear ribonucleoprotein family member FUS to RNA prevents R-loop RNA:DNA hybrid structures
Source: J Biol Chem. 2023 Sep 9;299(10):105237. doi: 10.1016/j.jbc.2023.105237 (PMC10556777; doi:10.1016/j.jbc.2023.105237)
Supplement: Supporting information [file mmc1.pdf]

Table S1

flag-FUS-BSU36I RNA	AGACCCAAGCUUGACCACCAUGGGUACCAUGCCAAAGAAGAAGAGG<br>AAGGUACCGAGCUCGGAUCCAAUGGACUACAAGGACGACGAUGACA<br>AAAUGGCCUCAAACGAUUAUACCCAACAAGCAACCCAAAGCUAUGG<br>GGCCUACCCCACCCAGCCCGGGCAGGGCUAUUCCCAGCAGAGCAG<br>UCAGCCCUACGGACAGCAGAGUUACAGUGGUUAUAGCCAGUCCAC<br>GGACACUUCAGGCUAUGGCCAGAGCAGCUAUUCUUCUUAUGGCCA<br>GAGCCAGAACACAGGCUAUGGAACUCAGUCAACUCCCCAGGGAUAU<br>GGCUCGACUGGCGGCUAUGGCAGUAGCCAGAGCUCCCAAUCGUCU<br>UACGGGCAGCAGUCCUCCUACCCUGGCUAUGGCCAGCAGCCAGCU<br>CCCAGCAGCACCUCGGGAAGUUACGGUAGCAGUUCUCAGAGCAGC<br>AGCUAUGGGCAGCCCCAGAGUGGGAGCUACAGCCAGCAGCCUAGC<br>UAUGGUGGACAGCAGCAAAGCUAUGGACAGCAGCAAAGCUAUAAUC<br>CCCC
ssDNA	ATGAAAATAAAAACAGGTGCACGCATCCTCGCATTATCCGCATTAACG<br>ACGATGATGTTTTCCGCCTCGGCTCTCGCCAAAATCGAAGAAGGTAA<br>ACTG
siFUS-F<br>siFUS-R	CGGACAUGGCCUCAAACGAdTdT<br>UCGUUUGAGGCCAUGUCCGdTdT
SCR-F<br>SCR-R	GAUGCAGACAUUCAGGAUGdTdT<br>CAUCCUGAAUGUCUGCAUCdTdT

Supplemental Figure 1

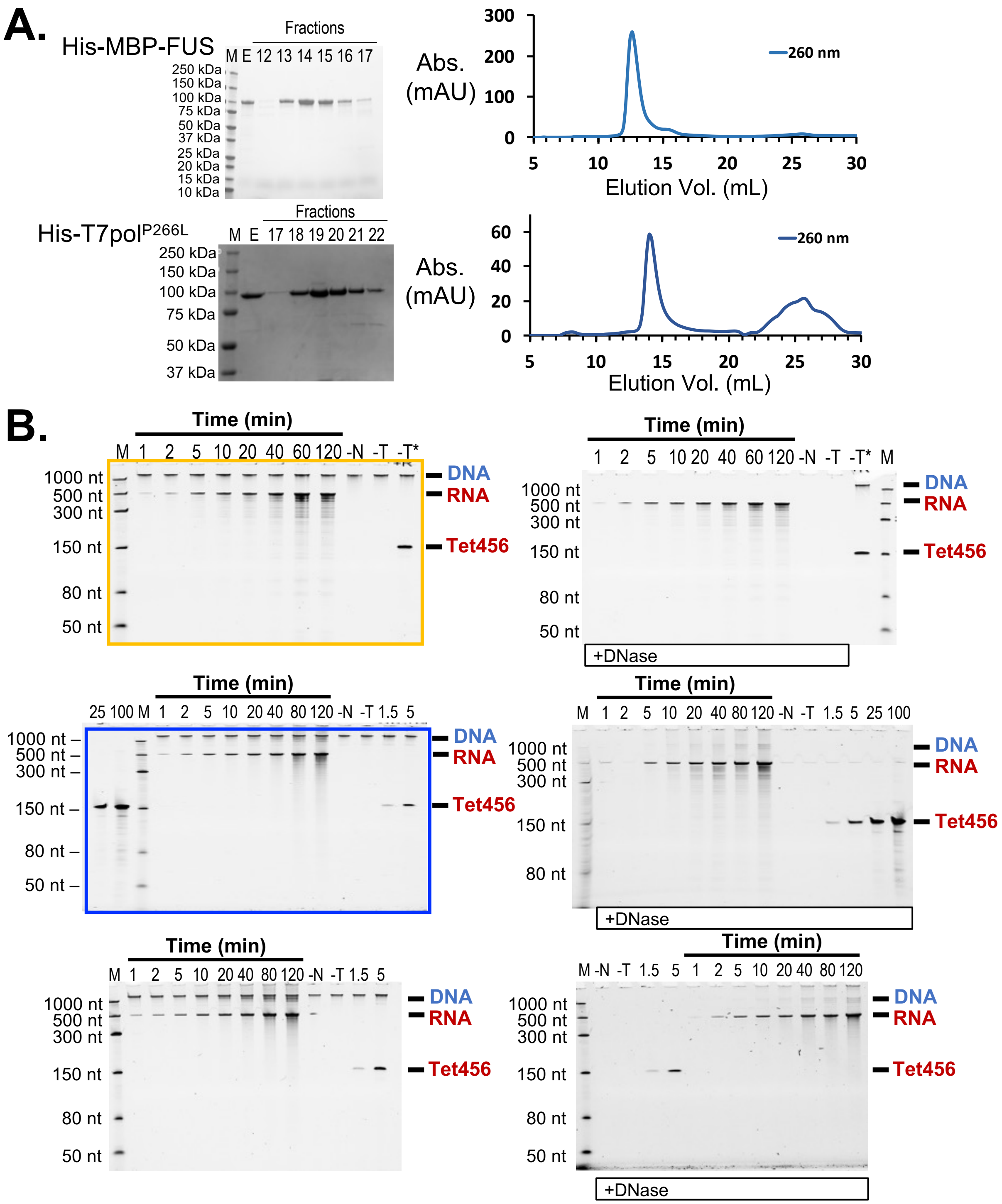

Supplemental Figure 1, *continued*

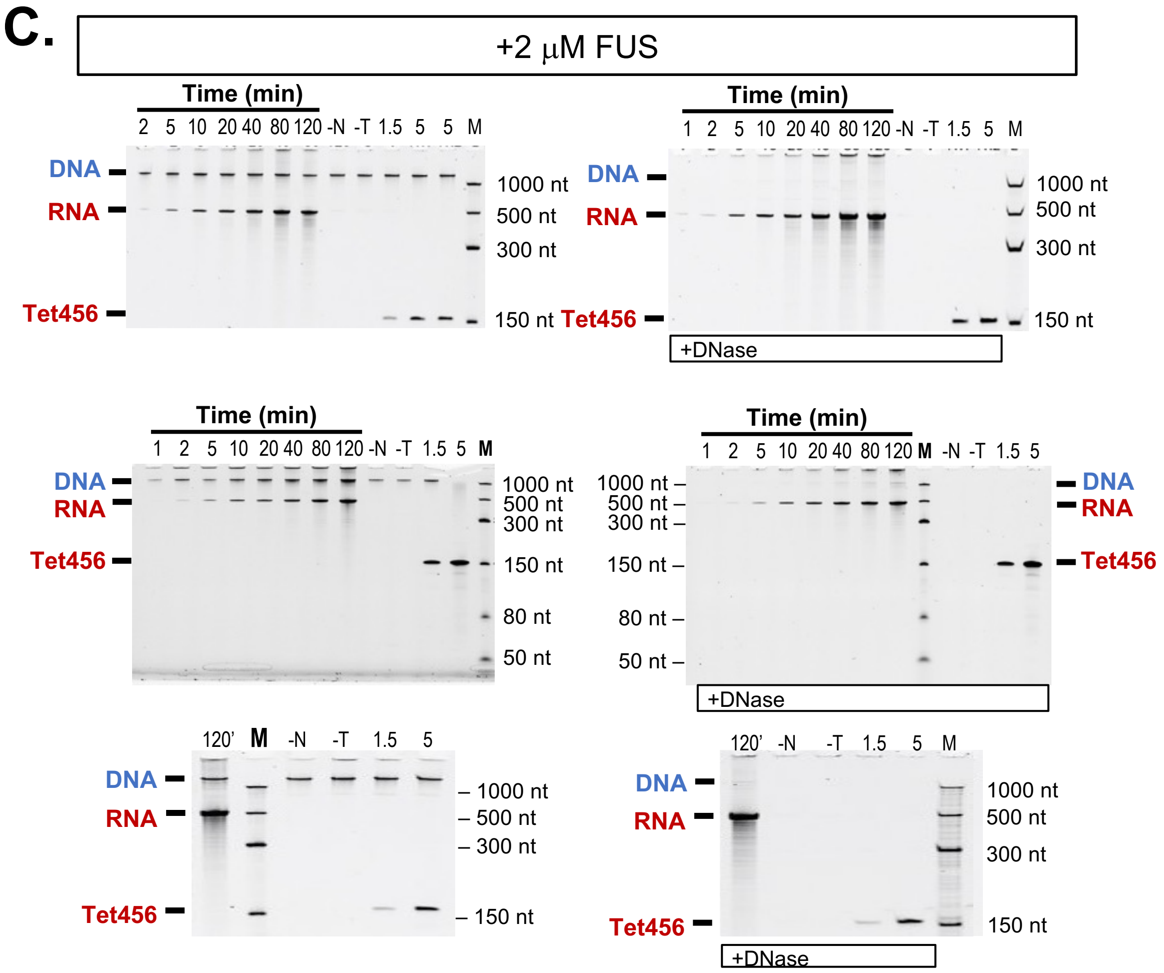

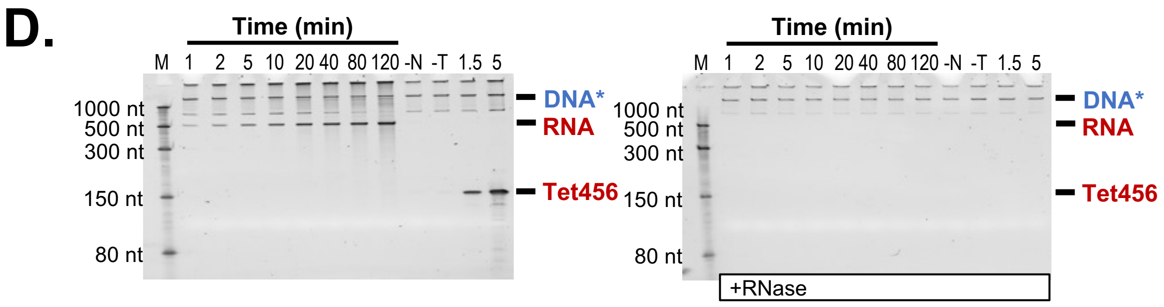

* An additional band seen between the RNA and linear DNA is circular plasmid, indicating an incomplete digestion by restriction enzyme.

Supplemental Figure 1, *continued*

E.

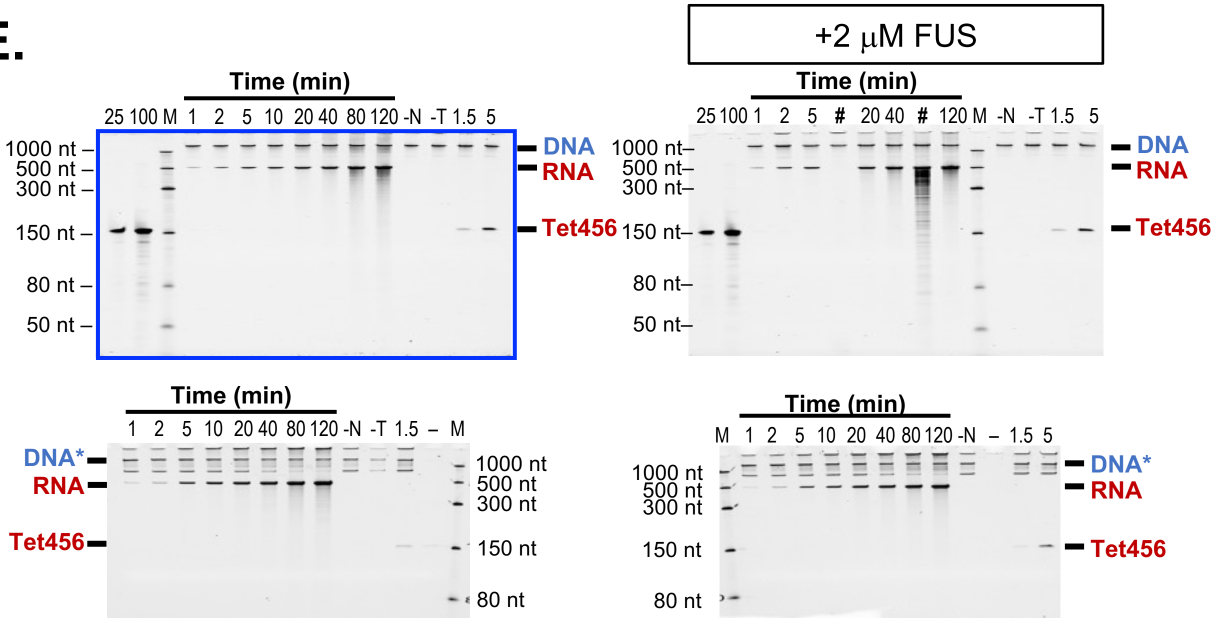

F.

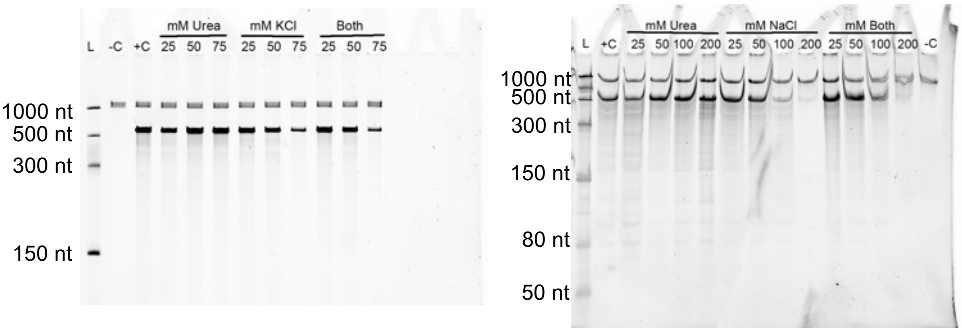

G.

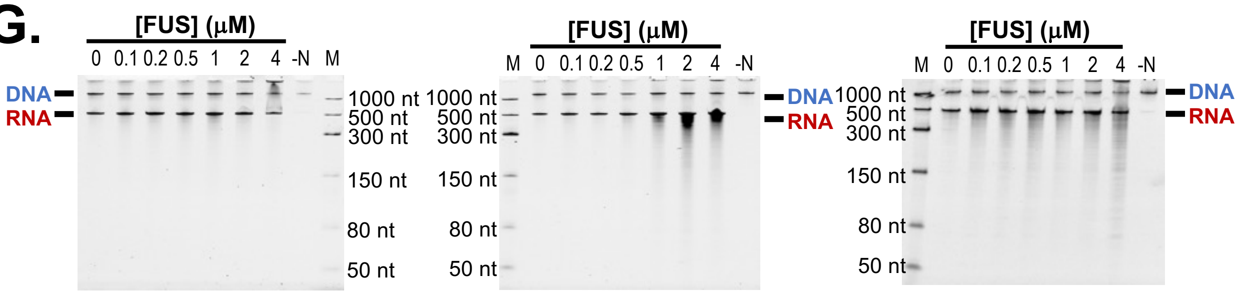

H.

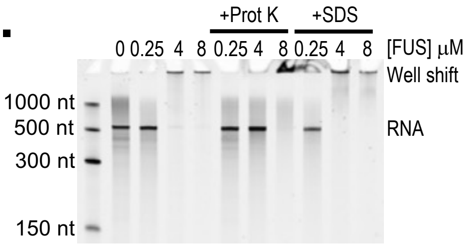

Supplemental Figure 1, *continued*

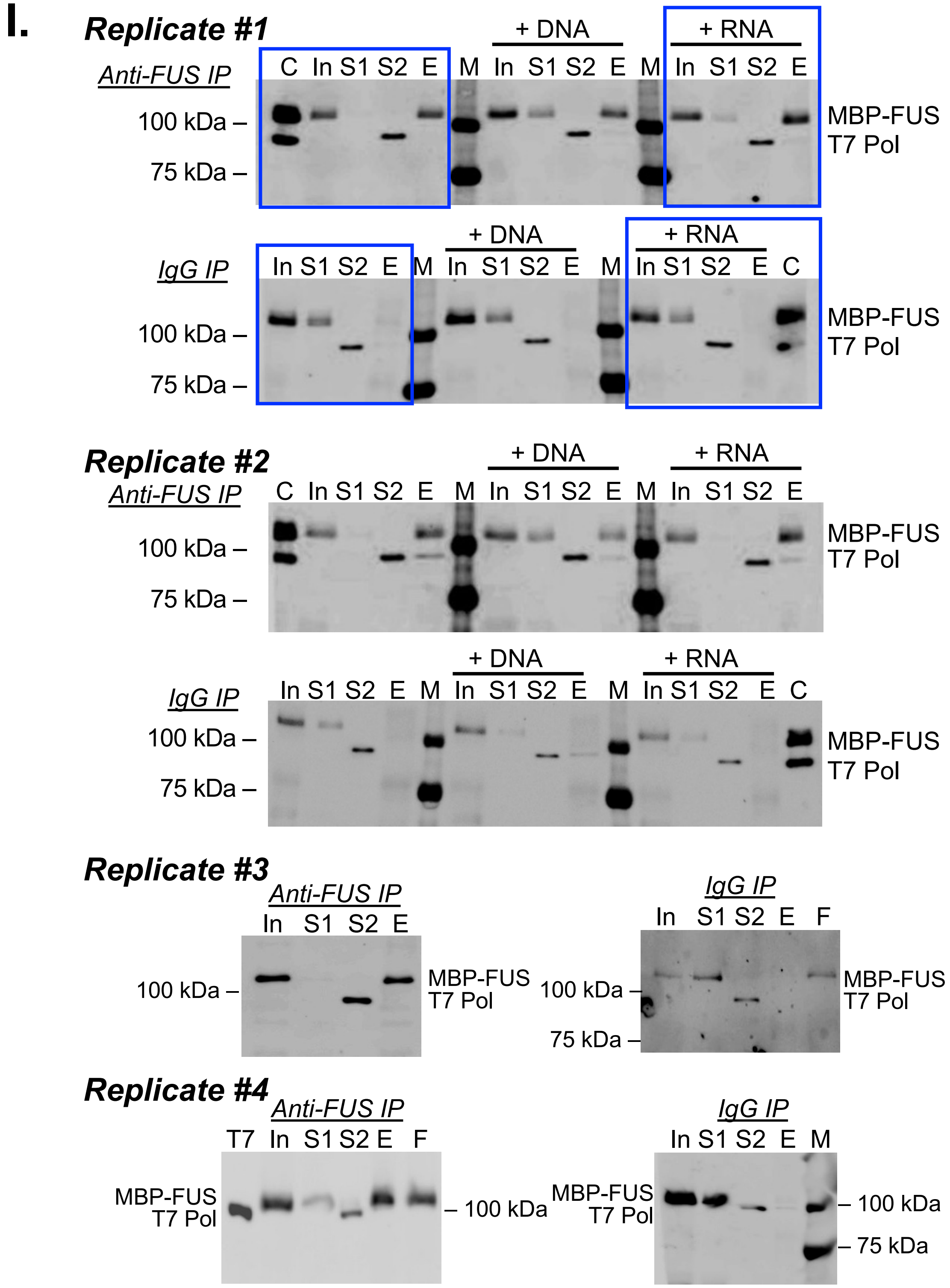

Supplemental Figure 1. Purification of protein and immunoprecipitation. (**A**) Coomassie stained SDS-PAGE gel, left, of fractions collected after separation by SEC (see UV absorption trace at 260 nm, right) of His-MBP-FUS and His-T7pol^{P266L} proteins. Following SEC, FUS proteins and T7 polymerase were tested for transcription activity and presence of nucleases before use. (**B**) Urea-PAGE gels stained with SYBR show the effect of DNase I on samples collected during T7 Pol transcription assays and (**C**) assays that have FUS (2 μM) present. Gels shown are untreated samples (left) and those following incubation with DNase I (right). Control samples were incubated for 120 minutes with NTPs (-N) or T7 Pol (-T) during the transcription assay. Lanes that include an RNA control (Tet456) are labeled by the amount in nanograms (ng) loaded to the gel. Lanes with 1.5 and 5 ng Tet456, RNA was included during the transcription assay. Lanes with 25 and 100 ng Tet456 show RNA loaded to the gel from stock. Asterisks (*) indicate samples without DNase treatment. The blue border in panel B indicates a gel seen also in S1E beside its counterpart performed with FUS present. The orange border in panel B indicates a gel seen also in S3A beside its corresponding dot blot assay. (**D**) Urea-PAGE gel images for samples collected during T7 Pol transcription (left) and those after treatment by RNaseA (right). (**E**) Replicates to Figures 1A and 1B: time course experiments for T7 Pol transcription without (left) or with (right) 2 μM FUS protein present. (#) indicates samples omitted from quantitation due to nuclease activity seen. (–) indicates lanes with no sample loaded. Controls are labeled as in (**B**). Gels bordered in blue are also included in panels B and C beside their counterpart treated with Dnase. (**F**) Urea-PAGE gel images of transcripts produced by T7 Pol in titrating concentrations of urea, KCl, or NaCl. (**G**) Urea-PAGE analysis with SYBR-staining of replicates for transcription assays with increasing concentration of FUS (replicate 4 shown in Figure 1C). (**H**) Testing the role of protein aggregation in shifting RNA to the well during PAGE analysis. Samples were treated with either proteinase K, Prot K, or sodium dodecyl sulfate, SDS. (**I**) Replicates of Co-IP of FUS with T7 Pol. Portions of replicate #1 indicated by blue squares are cropped and included in **Figure 1D**. Lanes loaded with MBP-FUS and T7 Pol protein from stock are labeled “C”. Lanes labeled “F” indicates loading MBP-FUS only. “M” indicates molecular weight ladder.

Supplemental Figure 2

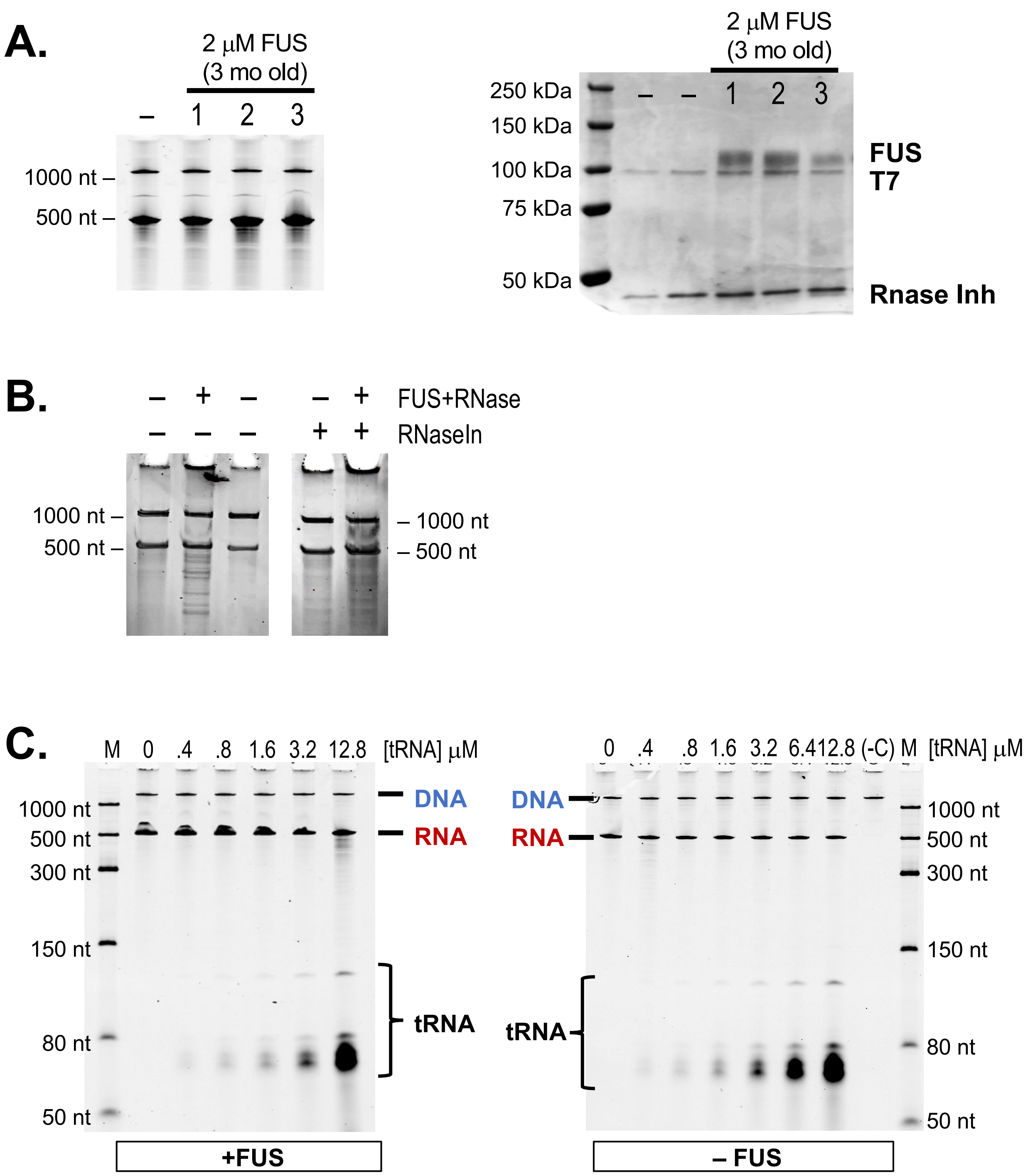

Supplemental Figure 2, *continued*

C. *(cont.)*

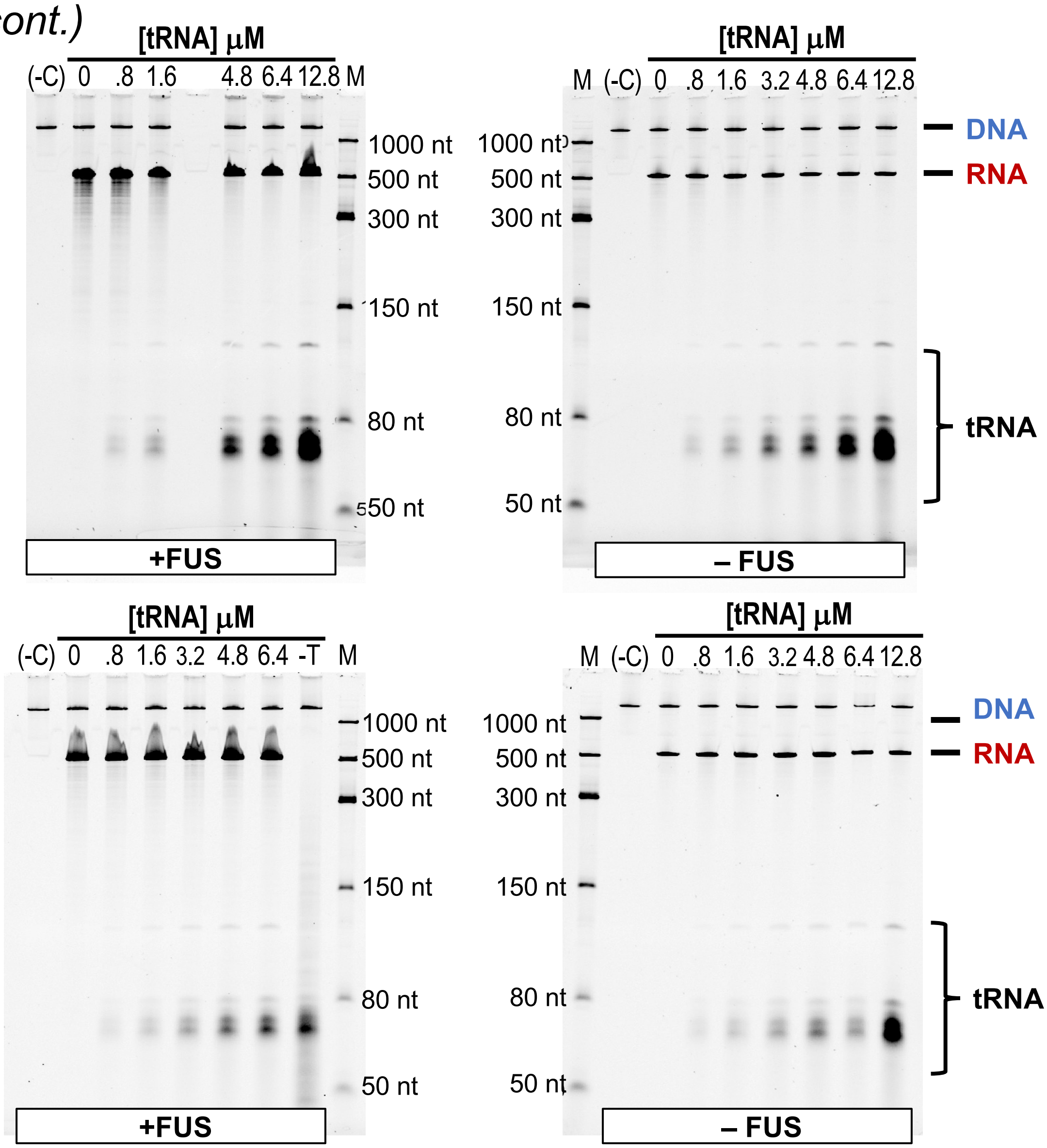

D.

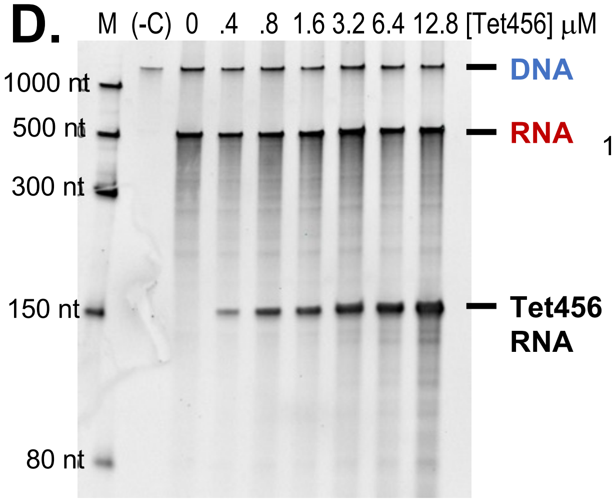

E.

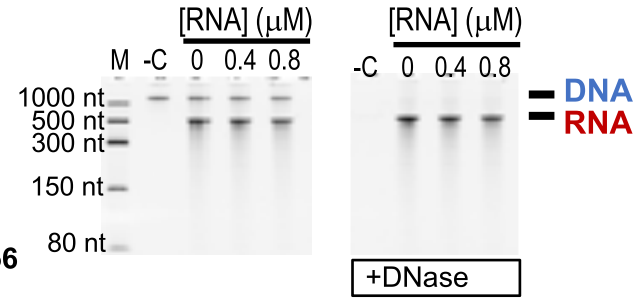

Supplemental Figure 2, *continued*

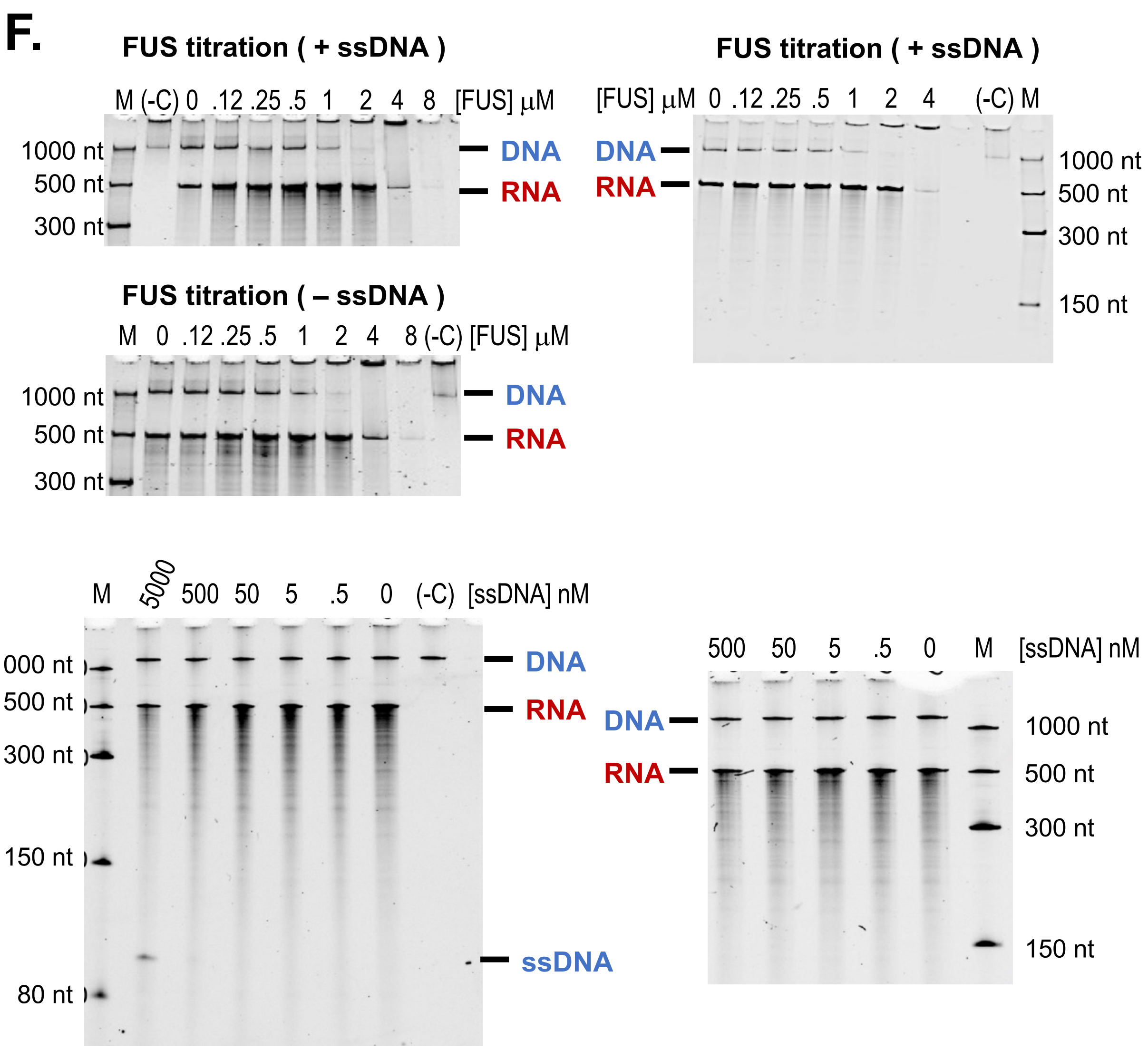

Supplemental Figure 2. FUS activity is not competed by RNA or DNA. (**A**) Assay of the same FUS preparations used for **Figure 1B and 1C**, re-assayed after 3 months of storage at room temperature. To the right are SDS-PAGE analysis stained by Coomassie for the same run-off transcription samples, showing full-length FUS protein (2 μM) was present in the transcription assays. (**B**) The effect of RNase inhibitor was tested on FUS protein co-purified with an RNAse. The inhibitor was able to block nuclease activity and activity of FUS (2 μM) could then be observed. (**C**) Titration of yeast tRNA into transcription run-off assays did not change the activity of FUS protein. Three replicate experiments are shown with 4 μM FUS (left) or no FUS (right) included in the reaction. (**D**) Titration of a TET456 RNA into transcription run-off assays did not change the activity of FUS protein. (**E**) Gel showing DNase treatment of samples with Tet456 RNA added. (**F**) Addition of single-stranded DNA, ssDNA, into transcription run-off assays did not change the titrated activity of FUS protein. Aggregation shifting DNA and RNA to the wells of the gel are observed at concentrations 2, 4, and 8 μM FUS for these assays. Also shown are control experiments titrating ssDNA into T7 Pol transcription assays. In all figure parts, control samples include omission of NTPs (-C) or T7 Pol (-T) from the assay.

Supplemental Figure 3

A.

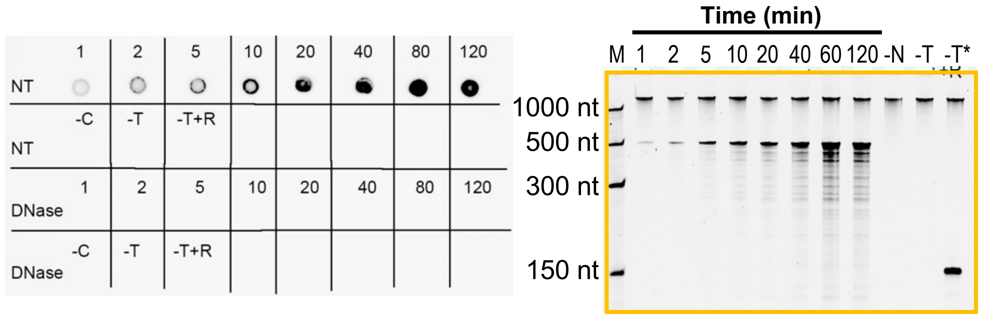

B.

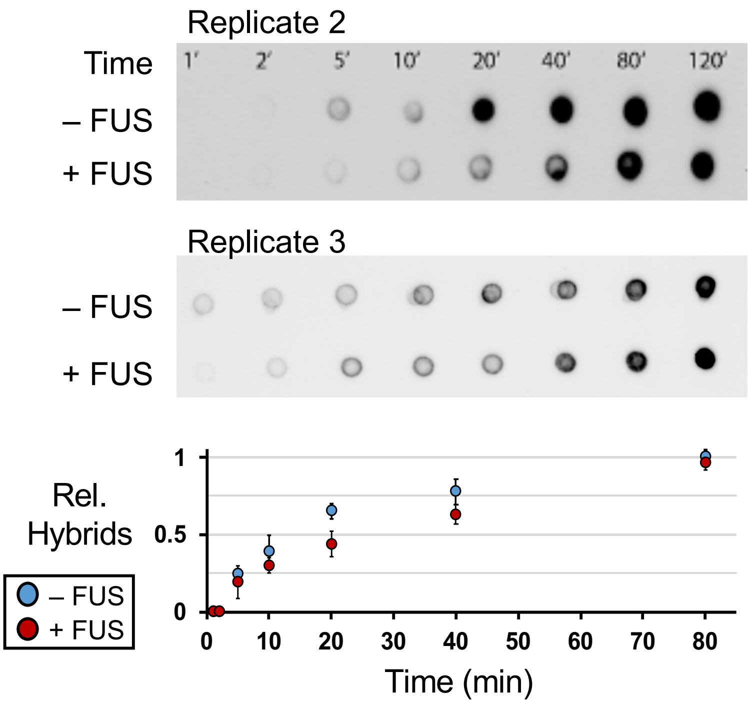

C.

[FUS] (µM)	0	0.12	0.25	0.5	1	2	4	8
**Replicate 1**	1.00	1.18	1.08	0.99	0.91	0.83	0.61	0.44
**Replicate 2**	1.18	0.86	0.94	0.97	0.93	0.82	0.50	0.62
**Replicate 3**	0.78	0.89	0.80	0.71	0.71	0.66	0.57	0.39

Supplemental Figure 3. Time course of RNA:DNA hybrid formation during run-off transcription assays. (**A**) Assay as shown in **Figure 3A** and showing controls of omitting NTP, (–), or T7 Pol, -Pol, or addition of a non-specific RNA, +R. Samples were incubated with Dnase I to remove DNA and show the S9.6 antibody requires both RNA and DNA strands to bind. Note: enhanced Dnase I activity toward RNA:DNA hybrids was achieved by increased concentrations of $MgCl_2$ (8 mM) and $CaCl_2$ (1.3 mM). RNA products were detected by PAGE analysis and Sybr staining. The additional band in the leftmost lane, -Pol+R, is that of TET456 RNA. The orange border indicates a gel seen also in S1B beside its corresponding gel showing effects of Dnase I. (**B**) Replicates of dot blot assay shown in **Figure 3A** showing hybrids formed during T7 Pol run-off assays in the absence or presence of FUS (2 μM). Quantification of dot blot assays show hybrid levels differed most at timepoints collected at 20 and 40 min. Results are averaged from 3 experiments. Error bars represent standard error about the mean (±SEM). (C) Table showing the densitometry measurements as adjusted volume units ($x10^6$) for replicates plotted in **Figure 3C**.

Supplemental Figure 4

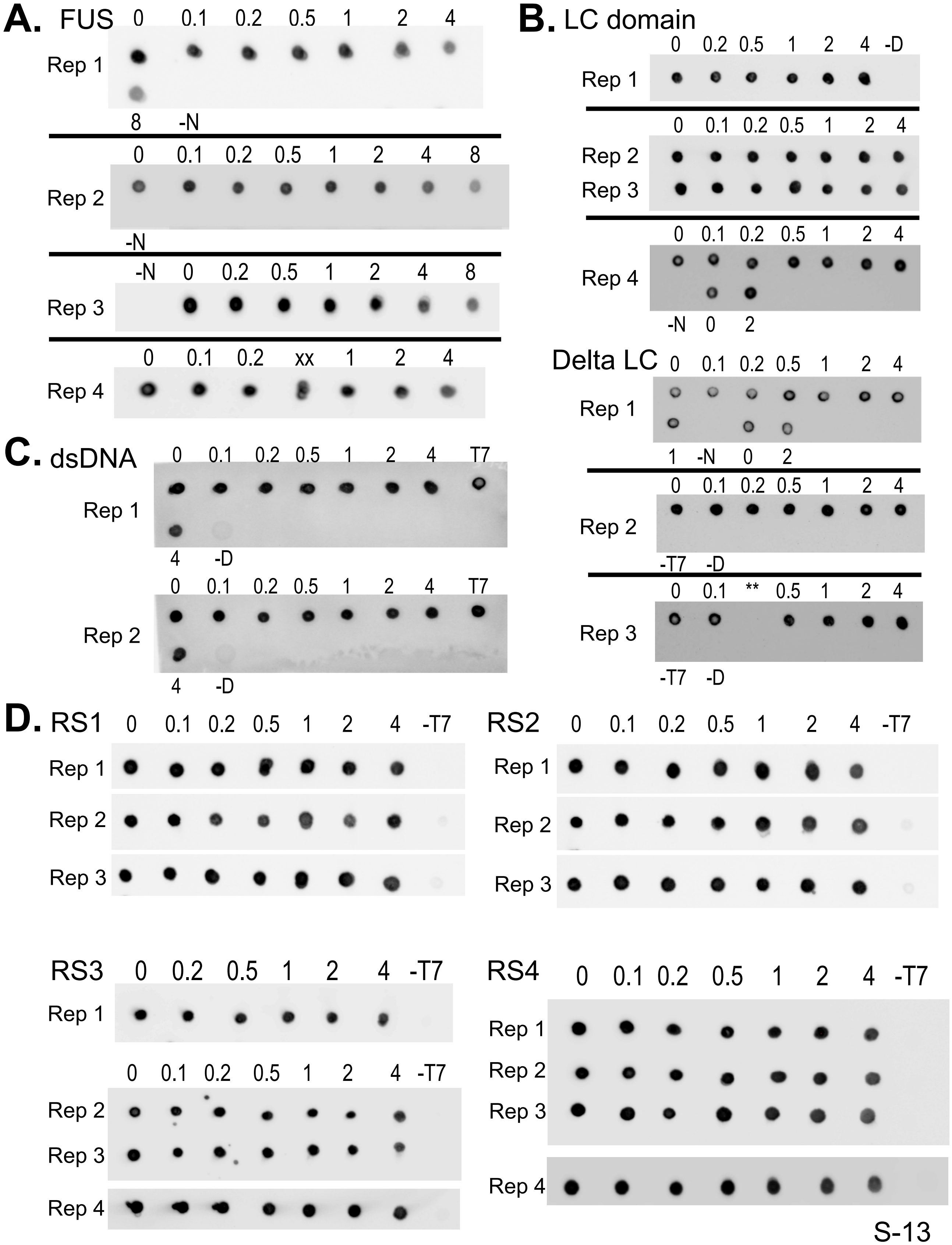

Supplemental Figure 4, *continued*

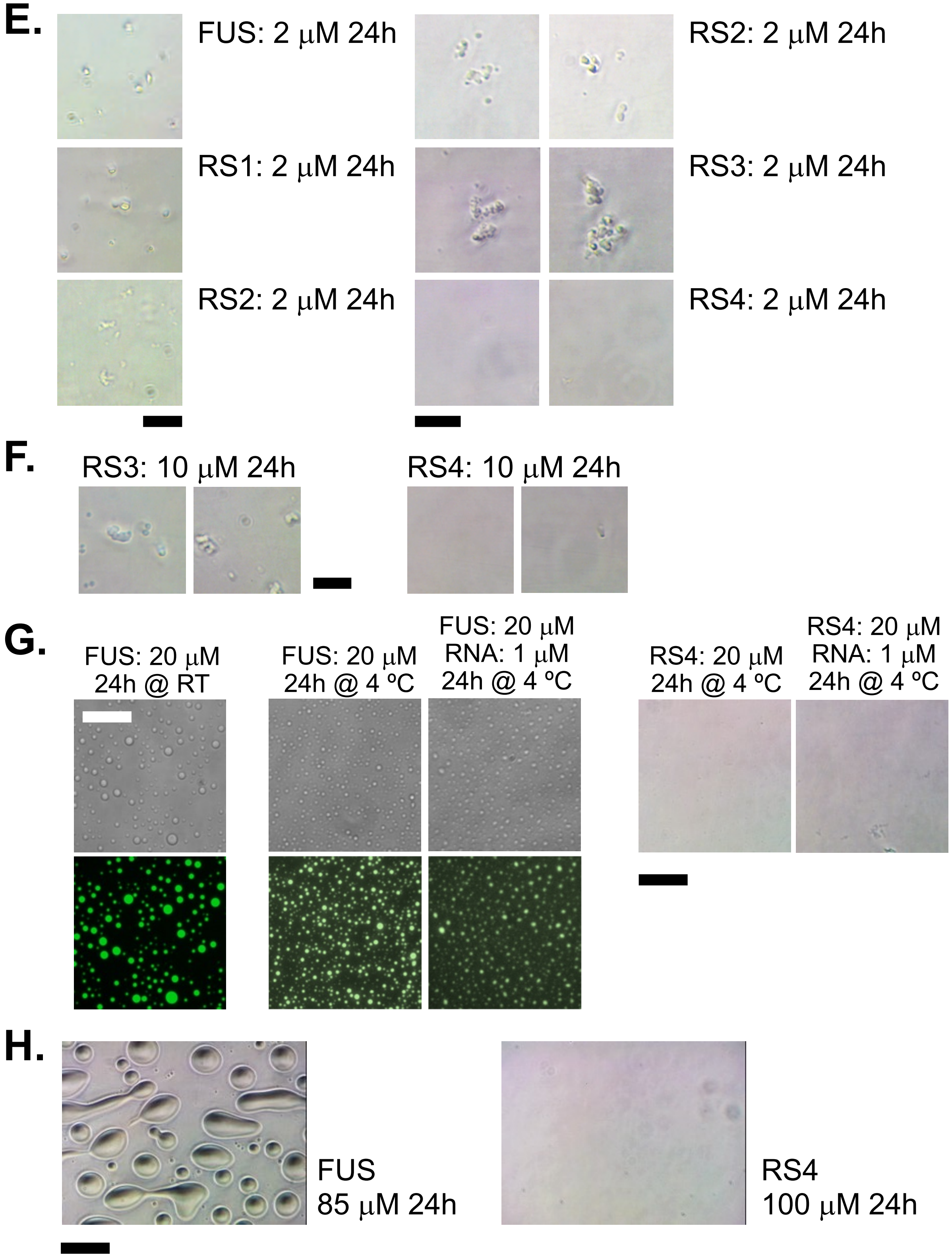

Supplemental Figure 4. Replicate dot blot assays for RNA:DNA hybrid formed during run-off transcription assays and inspection of FUS particles using phase contrast microscopy. (**A**) Replicates of assays with titrating concentrations of FUS. Replicate 3 is included in **Figure 3B**. (**B**) Dot blot assays for FUS-LC and Delta LC titrations. (**C**) Dot blot assays for FUS-LC titrations using an antibody to double-stranded DNA (dsDNA). (**D**) Dot blot assays for arginine substituted FUS mutants RS1, RS2, RS3, and RS4. (**E**) Particles assembled after 24-hour incubation of FUS or RS proteins (2 μM) in transcription run-off assay conditions. (**F**) Imaging for particles formed by RS3 and RS4 proteins at 10 μM, showing no clear evidence of RS4 assembly. (**G**) Imaging for particles formed by FUS and RS4 proteins (20 μM) after incubation for 24 hours at 4 ºC. Incubations of FUS and RS4 was repeated with addition of TET456 RNA (0.5 μM). Fluorescent images were made by addition of GFP-tagged FUS LC-domain (amino acids 1 to 266) at x150 lower concentration that FUS. (**H**) Imaging for particles formed by FUS or RS4 at high protein concentration as indicated. Scale bars are 2 μm for (**E**) and (**F**) and 4 μm in parts (**G**) and (**H**).

Supplemental Figure 5

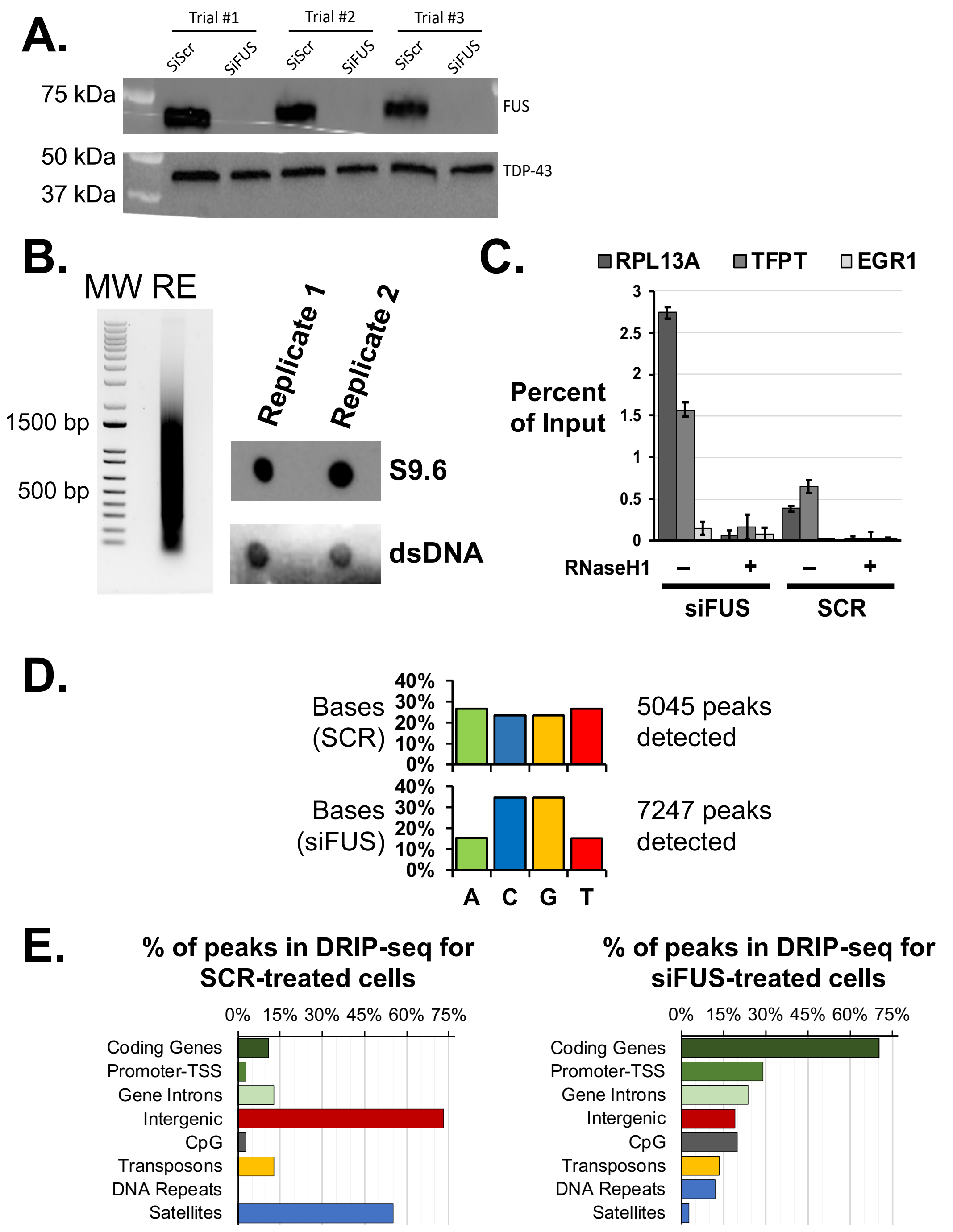

Supplemental Figure 5, *continued*

F.

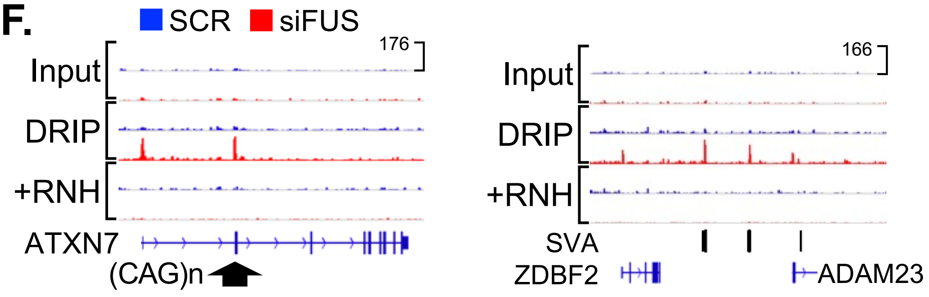

G.

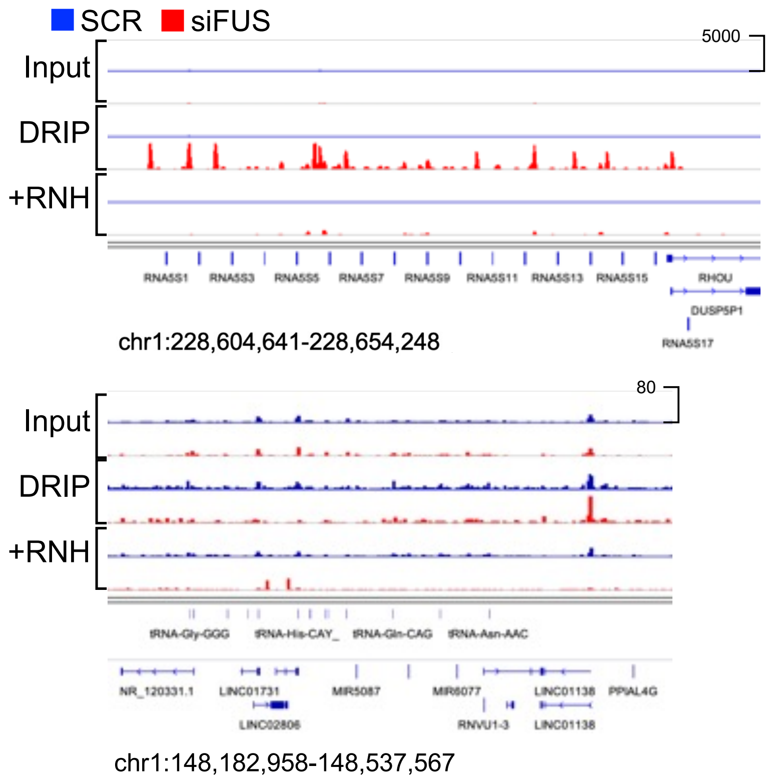

Supplemental Figure 5. DRIP-seq analysis of FUS knockdown in HEK293T/17 cells. (**A**) Western assay to confirm knockdown of FUS protein by siFUS relative to control, SCR, in HEK293T/17 cells, with TDP-43 protein serving as loading control. See also **Figure 5.** (**B**) Verification of restriction enzyme digestion of genomic DNA recovered from HEK293T/17 cells, left, and the presence of RNA:DNA hybrids in genomic DNA recovered by dot blot assay, right, using the S9.6 antibody with antibody to dsDNA as loading control. (**C**) Realtime PCR to detect RNA:DNA hybrids after immunoprecipitation for R-loop positive promoters RPL13A and TFPT, and R-loop negative promoter EGR1. Samples were also treated with Rnase H, +RNH, prior to pulldown as negative control. Results shown are from 2 replicates and error bars indicate standard error about the mean (±SEM). (**D**) The base composition of DNA sequences in peaks called by MACS2 for DRIP-seq of SCR or siFUS treated cells. A GC content of >60% was found in peaks called for siFUS-treated samples. (**E**) Protein-coding genes comprised the largest percentage of peaks detected in the FUS knockdown results, in contrast to SCR-treated sample results. (**F**) DRIP-seq analysis of siFUS-treated samples reveals a peak in R-loop signal arising at a CAG-repeat DNA sequence at the *ATXN7* gene and SVA sequences, a SINE retrotransposon sub-type. (**G**) DRIP-seq signals observed at a 5S rRNA gene cluster and a tRNA gene cluster, both located on chromosome 1.
